# Predicting treatment adherence in patients with drug-resistant tuberculosis: insights from socioeconomic, demographic, and clinical factors of patients in the rural Eastern Cape

**DOI:** 10.3389/ftubr.2025.1659333

**Published:** 2025-10-01

**Authors:** Lindiwe Modest Faye, Mojisola Clara Hosu, Ntandazo Dlatu, Joshua Iruedo, Teke Apalata

**Affiliations:** ^1^Department of Laboratory Medicine and Pathology, Faculty of Medicine and Health Sciences, Walter Sisulu University, Mthatha, South Africa; ^2^Department of Public Health, Faculty of Medicine and Health Sciences, Walter Sisulu University, Mthatha, South Africa; ^3^Department of Family Medicine, Walter Sisulu University, Mthatha, South Africa

**Keywords:** clinical factors, drug-resistant tuberculosis, treatment adherence, socioeconomic factors, predictive modeling, random forest model

## Abstract

**Background:**

Drug-resistant tuberculosis (DR-TB) poses a serious challenge to global health. Patients must follow complex medication regimens over long periods, and any failure to comply with these treatment plans can result in treatment failure, higher mortality rates, and an increased risk of developing additional drug resistance.

**Setting:**

The study was conducted in the rural Eastern Cape.

**Aim:**

This study aims to identify the key factors influencing treatment adherence among patients with DR-TB. Furthermore, it rigorously evaluates the predictive accuracy of machine learning models in assessing treatment adherence, with a strong focus on socioeconomic, demographic, and clinical factors.

**Methods:**

A retrospective analysis was conducted on patients with DR-TB. Data were collected from medical records. Four different models were developed and tested to evaluate their effectiveness in predicting treatment adherence: Random Forest, Logistic regression, Support Vector Machine (SVM), and Gradient Boosting.

**Results:**

The Random Forest model achieved an accuracy of 53.3% in predicting treatment adherence. An analysis of feature importance indicated that age, income, education, social history, patient category, and comorbidities were the most significant factors influencing adherence. Patients with higher incomes, higher levels of education, and fewer comorbidities were more likely to follow their treatment plans.

**Conclusion:**

Adhering to treatment for DR-TB involves a range of socioeconomic and clinical factors. Income, education level, and pre-existing health conditions significantly influence how well patients follow their prescribed treatment regimens. Understanding these influences is crucial for enhancing treatment outcomes and facilitating patients' journey toward improved health.

**Contribution:**

These findings suggest that machine-learning models, especially Random Forest algorithms, can effectively support clinical decision-making by identifying patients at risk of non-adherence to their treatment.

## 1 Introduction

Drug-resistant tuberculosis (DR-TB) poses a significant global health challenge, necessitating that patients adhere to complex, long-term medication regimens (1, 2). Despite advancements in treatment options, non-adherence remains a substantial barrier, particularly in low-resource settings, leading to increased morbidity, mortality, and ongoing transmission (3). In response to these challenges, the World Health Organization (WHO) launched the End TB Strategy, which emphasizes patient-centered care, integrated treatment approaches, and the provision of social support systems to enhance adherence and improve outcomes. This is particularly crucial in rural, high-burden areas, such as the Eastern Cape province of South Africa, where patients often face layered socioeconomic and healthcare access barriers (4–6). This study aims to identify key determinants of treatment adherence among DR-TB patients in such settings and evaluate predictive modeling approaches to support more targeted intervention strategies. However, the relative impact of these factors on DR-TB populations has not been thoroughly investigated (7). According to the WHO, treatment adherence for tuberculosis (TB) refers to the extent to which the prescribed medication regimen is followed (8). In 2017, an estimated 10 million individuals had active TB, with 9% of these cases involving people living with HIV (PLWH). Four years later, there was an increase with an estimated 10.6 million people (95% CI: 9.9–11 million) developing TB worldwide, equivalent to an incidence of 134 (95% CI: 125–143) per 100,000 population. This revealed an increase in TB incidence globally by 4.5%, reversing a long-term trend of a moderate 2% annual decrease over the past decade (8). This situation places an excessive strain on healthcare systems, as the lengthy treatment regimen increases the likelihood of poor treatment adherence, which can lead to the development of medication resistance. Patients with multiple infections who miss scheduled clinic appointments are more susceptible to poor treatment adherence and worse health outcomes (9–13). Due to this therapeutic challenge, treatment for HIV and TB is often interrupted, prolonging treatment durations and heightening the risk of drug resistance (14).

South Africa contributes about 20% of the global burden of TB/HIV co-infections, making it one of the countries most affected by the intertwined epidemic, with ~180,000 incident TB cases comprising people with HIV co-infection (15, 16). Consequently, many South Africans co-infected with HIV and TB require simultaneous treatment for both conditions (16). The dual challenges presented by both infections complicate adherence to treatment. It is well established that maximizing treatment adherence significantly improves the likelihood of successful TB treatment (17). This principle similarly applies to HIV infection, where sustained viral suppression depends on a high level of adherence to antiretroviral therapy (ART). Improving adherence can lead to reductions in HIV transmission and an enhancement in the quality of life for infected individuals (18, 19). Despite advancements in treatment availability and accessibility, TB/HIV co-infection continues to be a complex public health issue contributing to the inability of TB-control initiatives, especially in high-burden nations, to meet effective treatment goals (16, 20). This situation necessitates a new strategy in South Africa with a high prevalence of HIV/AIDS. Methods for evaluating patient compliance with anti-TB medication typically focus on the percentage of patients who take 80% or more of their prescribed doses. Both direct and indirect measures of adherence to anti-TB regimens have been proposed. Direct measurement can involve testing for drug metabolites in the blood or urine. Indirect measurement may include assessing pharmacy refill records every month or utilizing patient-reported outcome measures, such as the TB Medication Adherence Scale (TBMAS) (21–25).

Evaluating the factors that influence treatment adherence in patients with TB therapy, TB/HIV, and HIV individuals is essential for identifying effective health system interventions to support individuals undergoing ART and combined therapy for improved treatment outcomes. This study aimed to identify key factors that affect treatment adherence in DR-TB patients in the poor rural Eastern Cape. Additionally, it sought to develop a predictive model using random forest algorithms to estimate the likelihood of adherence based on socioeconomic, demographic, and clinical variables.

## 2 Research methods and design

### 2.1 Study population and sampling

The study population comprised all patients diagnosed with DR-TB and receiving treatment at selected high-burden rural clinics in the rural Eastern Cape between January 2018 and December 2020 (*N* = 450). A total of 108 patients were included using proportionate stratified sampling, ensuring representation across age groups, gender, and patient categories (new, relapse, TAL, TF1, TF2). The sample size (*n* = 108) was determined using the finite population correction formula for a known population (*N* = 450), assuming an expected adherence prevalence of 24% from prior literature, a 95% confidence level (*Z* = 1.96), and a precision (margin of error) of 5%. This yielded a minimum required sample size of 108 patients, which was then proportionately stratified by age, gender, and patient category to ensure representativeness of the study population. While the sample reflects the demographic and clinical composition of DR-TB patients in the participating clinics, the representativeness is limited to similar rural, high-burden settings within the rural Eastern Cape. As income data were not directly available, occupational categories served as socioeconomic proxies, which may limit comparability to other regions. Inclusion criteria were diagnosis of DR-TB between January 2018 and December 2020 at one of the participating high-burden rural clinics in the Eastern Cape; initiation on a standardized DR-TB treatment regimen; and availability of complete treatment records for the study period. Exclusion criteria included transfer out before treatment completion, loss to follow-up within the first month of treatment, incomplete or missing clinical/socioeconomic data, and being < 18 years of age. From the eligible population, a proportionate stratified sampling approach was used to ensure representation across age, gender, and DR-TB patient categories. While the sample reflects the composition of DR-TB patients in this rural setting, the findings may not be directly generalized to urban or low-burden regions.

### 2.2 Variables and definitions

Income category was used as a proxy for socioeconomic status (SES) and was not recorded as a direct numeric value. It was inferred from documented occupation in patient records and classified as follows: low income for those unemployed or employed in informal/manual labor; middle income for those employed in government positions or permanent skilled work; and high income for those employed in corporate/professional sectors or self-employed with formal business registration. Data were randomly partitioned into training (80%) and testing (20%) subsets, stratified by adherence status to maintain the class distribution. To ensure reproducibility, the random split was performed before partitioning. All model training and tuning were conducted on the training set only, with final performance metrics calculated on the held-out test set. All statistical analyses were performed using R statistical software, Version 4.5.1 (56).

#### 2.2.1 Microbiological definition of DR-TB and diagnostic workflow

All DR-TB cases in this cohort were laboratory-confirmed. Initial testing was performed with Xpert MTB/RIF (Ultra) on sputum or other respiratory spemens. Specimens with rifampicin resistance (RR) on Xpert triggered reflex line probe assays (LPAs) MTB DRplus v2 (for isoniazid/rifampicin) and MTBDRsl (for fluoroquinolones and second-line agents), and culture-based drug susceptibility testing (DST) using standard liquid culture systems. Where feasible, DST included bedaquiline and linezolid to align with contemporary Group A agents. Patient category labels (New, Relapse, TAL, TF1, TF2; see Section 2.4) describe treatment history only and were not used as the sole basis for DR-TB diagnosis.

Resistance category definitions (25):

RR-TB: resistance to rifampicin, detected by Xpert, LPA, or phenotypic DST, irrespective of isoniazid susceptibility.
MDR-TB: resistance to at least isoniazid and rifampicin.
pre-XDR-TB: MDR/RR-TB with additional resistance to any fluoroquinolone.
XDR-TB: MDR/RR-TB with additional resistance to any fluoroquinolone and at least one Group A drug (i.e., bedaquiline or linezolid).

Laboratory methods followed National TB Program procedures for specimen collection, processing, quality control, and external proficiency testing. Results from Xpert, LPAs, and culture-DST were reconciled in the medical record; when discordant, phenotypic DST and/or LPA guided final categorization.

The sample size calculation for the population was adapted from the work of Agresti and Finlay (26).


$$\begin{array}{l} Sample\;proportion\;\left(\%\right)=\frac{\text{Sample size }by}{Total\;population}X\;100 \end{array}$$

$$\begin{array}{l} Sample\;proportion\;\left(\%\right)=\frac{108}{450}X\;100 \end{array}$$

$$\begin{array}{l} Sample\;proportion\;\left(\%\right)=0.24 \end{array}$$

The confidence interval for a population proportion of 24% was established based on the estimation of the proportion. The sample size is *n* = 108, the total population is *N* = 450, and the observed proportion is:


$$\begin{array}{l} \text{p}=\frac{108}{450} \end{array}$$

$$\begin{array}{l} \text{p}=0.24\;\left(24\%\right) \end{array}$$

A confidence interval (CI) of 95% for a proportion was calculated as:


$$\begin{array}{l} CI=p\pm Z\times \sqrt{\frac{\text{p}\left(1-\text{p}\right)}{\text{n}}} \end{array}$$

where:

*p* = 0.24 (sample proportion),

*Z* = 1.96 for a 95% confidence level,

*n* = 450 (total population).

The 95% CI indicates that the proportion of 24% is estimated to lie within the range of 20.05% to 27.95%. The key variables examined included age (in years), gender (male, female), income level (low, middle, high), education (no formal education, primary, secondary, tertiary), HIV status (positive, negative), comorbidities (the number of concurrent health conditions), social history (smoking, drinking habit), occupation (unemployed, manual labor, government employee, corporate job), and patient classification (new cases, relapses, or chronic cases, such as TAL—treatment after loss to follow-up, TF1—treatment following failure of first-line drugs, and TF2—treatment after failure of second-line drugs).

### 2.3. Ethical consideration

Ethical approval for the study was obtained from the Walter Sisulu University Ethics Committee (Reference No. 026/2019) and formally approved by the Eastern Cape Department of Health (Reference No. EC_201904_011), following communication and submission of the study protocol to relevant provincial health authorities. Consent was deemed unnecessary, as the research involved using patient records. Self-reported adherence from patients is evaluated using a custom benchmark of 80% compliance. Adherence was calculated as the percentage of actual treatment days completed compared to the expected treatment days, using the following formula:


$$\begin{array}{l} Adherence\;\left(\%\right) \end{array}$$

$$\begin{array}{l} =\;\frac{Actual\;treatment\;days\;completed}{Expected\;treatment\;days}X\;100 \end{array}$$

The type of regimen considered in the study was classified into two categories: short regimens, which were assumed to last 6 months (180 days), and long regimens, which were set at 18 months (540 days). For each patient, we calculated adherence percentages and categorized them into adherent and non-adherent groups accordingly. We then summarized the proportions of adherent vs. non-adherent patients, providing a clear overview of compliance with the 80% benchmark. Clinical evidence indicates that adherence levels of 80% or higher are essential for achieving therapeutic success across various medical conditions.

### 2.4. Operational definition

In this study, drug-resistant TB (DR-TB) was defined microbiologically as RR-TB, MDR-TB, pre-XDR-TB, or XDR-TB per WHO/S.A. definitions (see Section 2.2.1). Patient categories (New, Relapse, TAL, TF1, TF2) reflect treatment history and were not used to define DR-TB in the absence of laboratory confirmation.

In the context of TB, the WHO defines adherence as how well a patient's actions follow the medical instructions for their treatment plan. Essentially, it's about whether a patient's behavior, such as taking medications on time and completing the full course, aligns with the prescribed regimen.
New patients are patients who have never been treated for TB or have taken anti-TB medications for less than 4 weeks.
Relapse patients are patients who were previously treated for TB, were declared cured or completed treatment at the end of their most recent course of treatment, and are now diagnosed with a recurrent episode of TB (either a true relapse or a new episode of TB caused by reinfection).
TAL are patients previously treated for TB who were declared lost to follow-up (LTFU) at the end of their most recent course of treatment.
TF1 are those patients who have previously been treated for TB with first-line drugs such as isoniazid, rifampicin, ethambutol, pyrazinamide, and streptomycin, and whose treatment failed at the end of their most recent course of treatment.
TF2 are those who have previously been treated for DR-TB with second-line drugs such as bedaquiline, linezolid, moxifloxacin, levofloxacin, clofazimine, cycloserine, para-aminosalicylic acid, propylthiouracil, and amikacin, and whose treatment failed at the end of their most recent course of treatment.

### 2.5. Model development

Among the four models tested, the Random Forest algorithm achieved the highest raw accuracy (81.5%) and balanced accuracy (53.3%), outperforming logistic regression (raw accuracy 79.6%, balanced accuracy 50.0%), gradient boosting (raw accuracy 78.7%, balanced accuracy ~49.5%), and SVM (raw accuracy 77.8%, balanced accuracy ~48.7%) (**Figures 3**, **4**, **Table 2**). Because the dataset was imbalanced (79.6% adherent vs. 20.4% non-adherent), reporting balanced accuracy was essential to evaluate performance across both classes. For comparison, a naive baseline classifier that predicted all patients as adherent achieved 79.6% raw accuracy but only 50.0% balanced accuracy, highlighting the modest yet meaningful improvement gained with Random Forest. Random Forest also demonstrated the best balance between precision and recall, reflected in its highest F1-score (0.59). These findings suggest that, while predictive performance was modest, Random Forest provided the most clinically balanced trade-off between identifying at-risk patients and avoiding excessive false alerts.

### 2.6. Training the model

A random forest classifier was developed using a comprehensive dataset that included a variety of socioeconomic, demographic, and clinical factors related to patients. This dataset featured crucial variables such as age, income, education level, gender, HIV status, existing comorbidities, and lifestyle factors like smoking and alcohol use. Thereafter, the dataset was carefully divided into training and testing subsets. This strategy allowed the classifier to learn and adapt from the training data while providing a means to assess its performance on a separate set of unseen data. This process is essential for verifying the model's ability to generalize its findings beyond the specific examples included in the training phase. The model's effectiveness was evaluated through various metrics, which helped to measure its accuracy and reliability in predicting outcomes based on the input features. This evaluation process is critical for ensuring the model's practical applicability in real-world scenarios.

### 2.7 Evaluating features of importance

The random forest model assesses the importance of each feature by examining its role in minimizing the model's prediction error. This evaluation process involves a detailed analysis of how the various decision trees within the random forest utilize different features to partition the data points effectively, thereby enhancing the accuracy of classifications. The importance score assigned to each feature is determined through an aggregation process that considers the frequency and effectiveness of each feature employed across the entire ensemble of trees. Features that play a significant role in diminishing impurity, measured by metrics such as Gini impurity or entropy, are recognized as more crucial for the model's predictive performance. This method ensures a comprehensive understanding of each feature's contribution to the overall model efficacy.

## 3 Results

### 3.1 Participant characteristics

Out of the 108 DR-TB patients included in the study, in Table 1 distribution of demographic, socioeconomic, and clinical characteristics for all study participants, frequencies (*n*) and percentages (%) are shown for each category. The largest age group was 20–30 years (24.1%), followed by 30–40 years (22.2%) and 40–50 years (17.6%). Males comprised 52.8% of participants. Most patients had low (41.7%) or middle income (35.2%), with only 23.1% in the high-income category. Education levels varied, with 29.6% having secondary education and 25.9% tertiary education. HIV prevalence was 50.9%. Comorbidities were present in 69.4% of patients, with 30.6% having two or more.

**Table 1:** Presents the detailed distribution of demographic and clinical variables.

**Variable**	**Category**	**Frequency (*n*)**	**Percentage (%)**
Age group (years)	20–30	26	24.1%
	30–40	24	22.2%
	40–50	19	17.6%
	50–60	17	15.7%
	60–70	12	11.1%
	≥70	10	9.3%
Gender	Male	57	52.8%
	Female	51	47.2%
Income level	Low	45	41.7%
	Middle	38	35.2%
	High	25	23.1%
Education level	No formal education	21	19.4%
	Primary education	27	25.0%
	Secondary education	32	29.6%
	Tertiary education	28	25.9%
HIV status	Positive	55	50.9%
	Negative	53	49.1%
Comorbidities	None	33	30.6%
	One	42	38.9%
	Two or more	33	30.6%
Smoking history	Smoker	40	37.0%
	Non-smoker	68	63.0%
Alcohol use	Alcohol use	36	33.3%
	No alcohol use	72	66.7%
Occupation	Unemployed	32	29.6%
	Manual labor	26	24.1%
	Government employee	25	23.1%
	Corporate job	25	23.1%
Patient classification	New	36	33.3%
	Relapse	30	27.8%
	TAL (Loss to follow-up)	12	11.1%
	TF1 (1st-line failure)	15	13.9%
	TF2 (2nd-line failure)	15	13.9%

Income level inferred from occupation: unemployed/manual labor = low; government employment = middle; corporate/professional = high. DR-TB categories (RR/MDR/pre-XDR/XDR) were assigned based on microbiological testing (Xpert, LPA, culture-DST) as per 2.2.1.

Patient category definitions: New = never treated for TB or < 4 weeks of treatment; Relapse = previously cured/completed treatment, now recurrent TB; TAL = treatment after loss to follow-up; TF1 = treatment failure after first-line drugs; TF2 = treatment failure after second-line drugs, as defined in the South African DR-TB Clinical Management Guidelines (55).

### 3.2. Treatment adherence patterns

The outcome adherence was operationalized from final treatment status as a binary outcome: adherent if the patient was cured or treatment completed, and non-adherent if lost to follow-up, treatment failed, or died. Records coded as moved out, transferred out, or still on treatment were excluded from regression analyses. We fit a multivariable logistic regression with adherence as the outcome (adherent = cured/completed; non-adherent = LTFU/failed/died; outcomes 6–8 excluded). Predictors included age group, sex, comorbidities (≥1 vs. none), smoking, alcohol use, smear positivity, long vs. short regimen at treatment start, and patient category (Relapse, TAL, TF1 vs. New). Age bands were collapsed (20–39, 40–49, ≥50) (26, 27). Missing data were handled by listwise deletion.

In Table 2, sex (OR = 14.36; 95% CI: 1.61–127.82; *p* = 0.017) and short regimen at start (reference; long regimen at start associated with lower odds of adherence: OR = 0.04; 95% CI: 0.00–0.30; *p* = 0.002) were significant. TAL (vs. New) (OR = 0.02; 95% CI: 0.00–0.41; *p* = 0.012) and TF1 (vs. New) (OR = 0.01; 95% CI: 0.00–0.41; *p* = 0.015) showed lower odds of adherence. Other covariates were not statistically significant in this specification. Reference categories included male, no comorbidity, non-smoker, no alcohol use, smear-negative, short regimen at start, age 20–39, and new patient category.

**Table 2:** Logistic regression of predictors of adherence among DR-TB patients (*n* = 90)

**Variable**	**OR**	**95% CI**	***p*-value**
Age 40–49 vs. 20–39	3.55	0.18–68.43	0.401
Age ≥ 50 vs. 20–39	1.35	0.12–15.19	0.809
Female (vs. male)	14.36	1.61–127.82	0.017
≥1 comorbidity (vs. none)	1.30	0.10–17.32	0.844
Smoking (yes vs. no)	12.71	0.73–221.49	0.081
Alcohol use (yes vs. no)	0.11	0.01–1.62	0.107
Smear positive (vs. negative)	0.30	0.05–1.96	0.211
Long regimen at start (vs. short)	0.04	0.00–0.30	0.002
Relapse vs. New	0.50	0.05–5.24	0.562
TAL vs. New	0.02	0.00–0.41	0.012
TF1 vs. New	0.01	0.00–0.41	0.015

Model statistics:

• *N* (included) = 90; pseudo-*R*^2^ (McFadden) = 0.522

• Likelihood ratio χ^2^(11) = 45.49, *p* = 3.98e−06

• Missing data handling: listwise deletion (no imputation).

### 3.3 Socioeconomic and occupational influences

Adherence improved progressively from unemployed patients (65%) to those in manual labor (72%), government employment (85%), and corporate roles (88%). Income and education were the variables most strongly associated with adherence in descriptive analyses.

### 3.4 Social and behavioral factors

Peer influence was linked to the lowest adherence (55%), followed by mental health issues (58%), unstable lifestyle (60%), alcohol use (65%), and smoking (62%). Demographics of women had a significantly higher adherence rate (75.4%) than men (58.8%). Adherence was highest in younger patients (85–88%) and declined with age, reaching its lowest point in older patients (6–65%). In socioeconomic factors, adherence improved with higher education and socioeconomic status. The rate increased from 62% for those with no formal education to 85% for those with tertiary education. Similarly, adherence was lowest for the unemployed (65%) and highest for corporate workers (88%). Health and social factors of HIV-negative individuals showed higher adherence (82%) compared to HIV-positive patients (68%). Social influences were also a factor; the lowest adherence rates were linked to peer influence (55%) and mental health issues (58%). Additionally, an unstable lifestyle (60%), smoking (62%), and alcohol use (65%) were all associated with lower adherence. 3.5 Adherence by patient category

**Figure 1:**
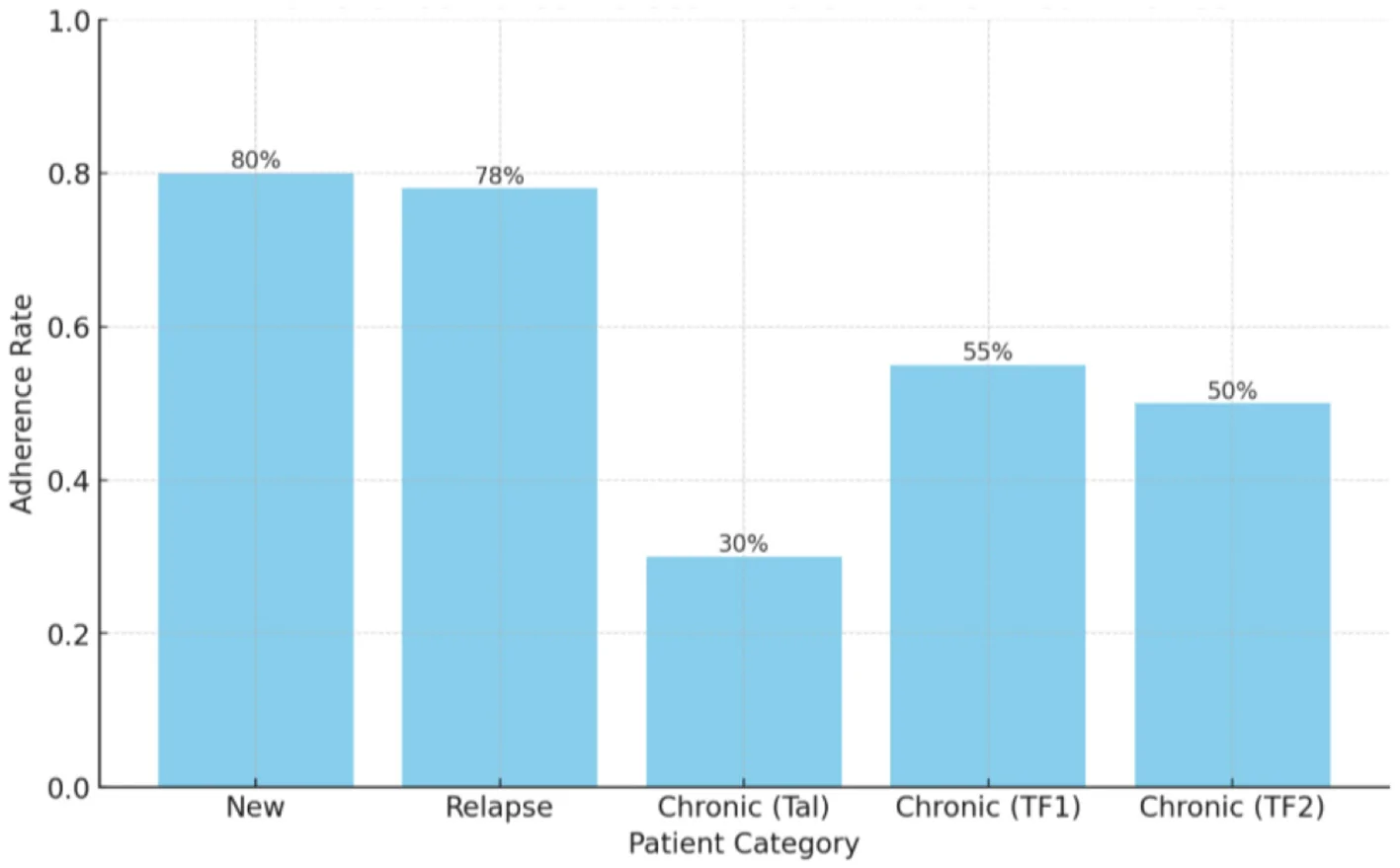
Comparison of adherence rates by patient classification type (patient categories). a = Categories defined per South African DR-TB treatment guidelines (see Table 1 footnote). b = Adherence calculated as actual treatment days completed ÷ expected treatment days × 100%.

New patients had the highest adherence (80%), followed by relapse cases (78%). Chronic patient categories had lower adherence, with TAL (30%), TF1 (55%), and TF2 (50%) showing the poorest performance (Figure 1). The chronic (TF1) and chronic (TF2) categories exhibit moderate adherence rates of 55% and 50%, respectively, indicating areas that still require improvement.

### 3.5 Variable associations

**Figure 2:**
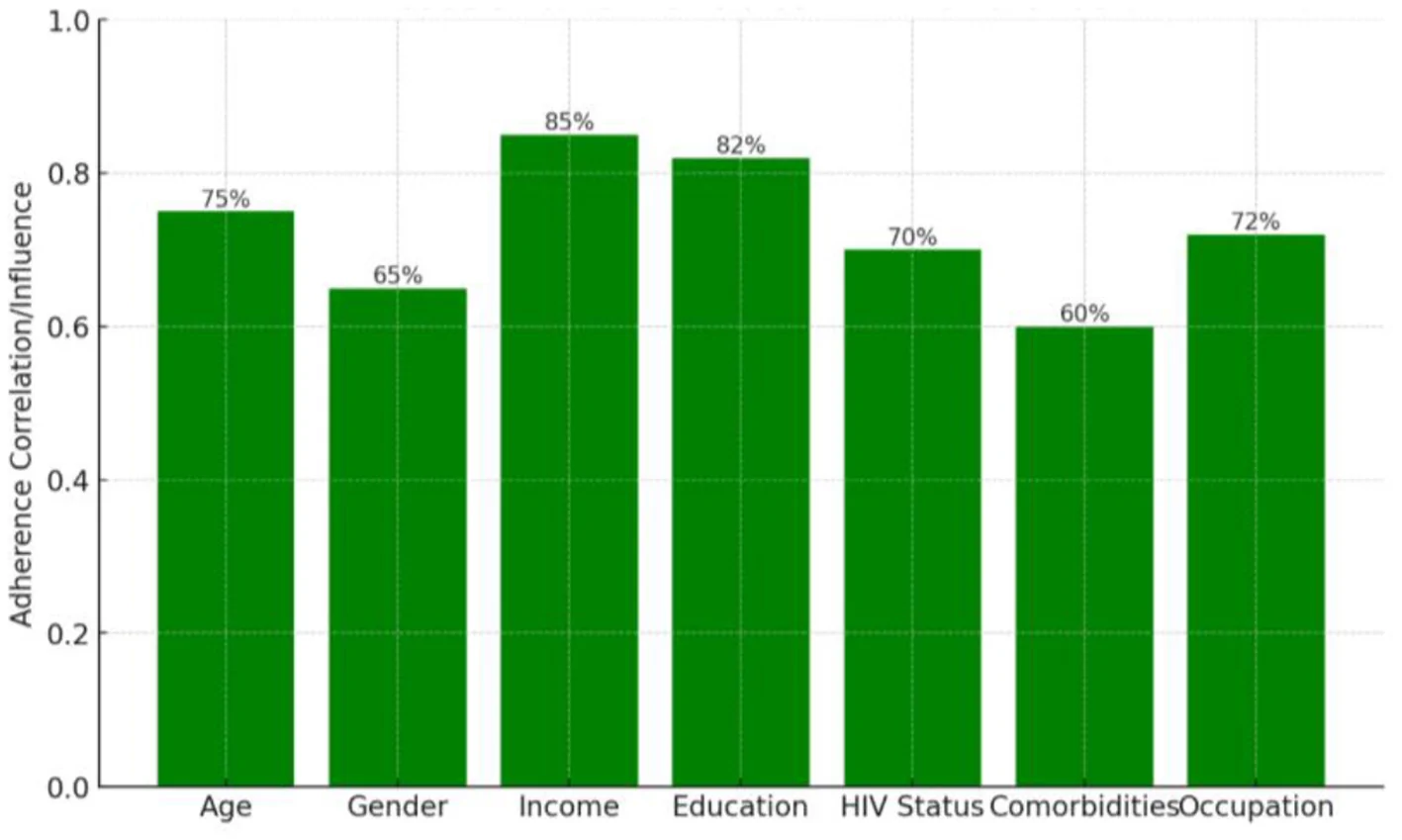
Strength of association between selected variables and treatment adherence, expressed as percentage association values. a = Associations are descriptive and not adjusted for confounding variables. b = Higher percentages indicate stronger observed relationships between the variable and adherence in this dataset.

Income (85%) and Education (82%) demonstrated the most substantial positive associations with adherence, suggesting that socioeconomic status and educational attainment are primary determinants of a patient's ability to follow their treatment regimen. Age and occupation also showed significant relationships with adherence, though their specific percentages were not provided in the summary. HIV status was negatively associated with adherence, indicating that patients who are HIV-positive faced greater challenges with treatment compliance. In contrast, gender (65%) and the presence of comorbidities (60%) showed weaker associations, suggesting they have a comparatively less influence on adherence compared to income and education (Figure 2).

### 3.6 Feature importance in predictive modeling

**Figure 3:**
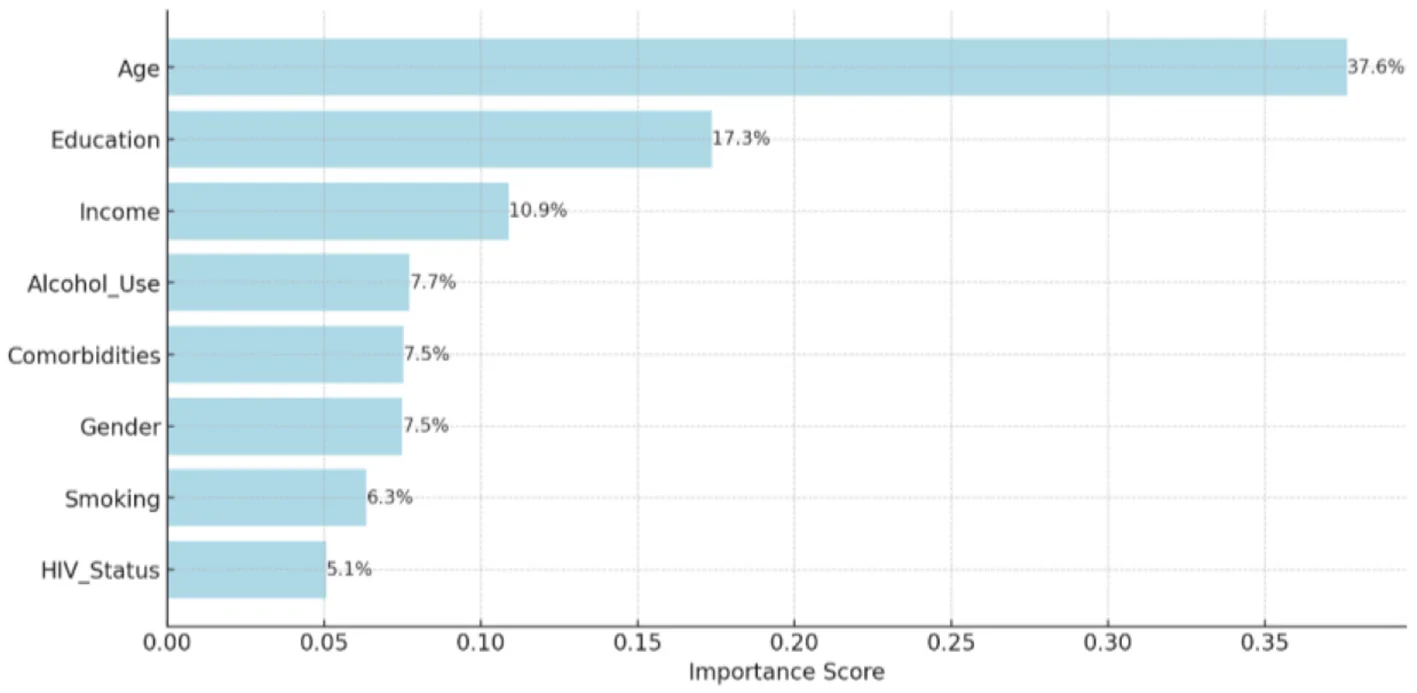
Relative contribution of each predictor variable to the Random Forest model's classification accuracy. a = Importance scores represent the mean decrease in Gini impurity across decision trees. b = Variables defined as in Table 1 footnotes a–c.

In the Random Forest model, age contributed the most to prediction (37.6%), followed by education (17.3%) and income (10.9%). Alcohol use, comorbidities, gender, and HIV status were of moderate to lower importance (Figure 3).

### 3.7 Model performance

**Figure 4:**
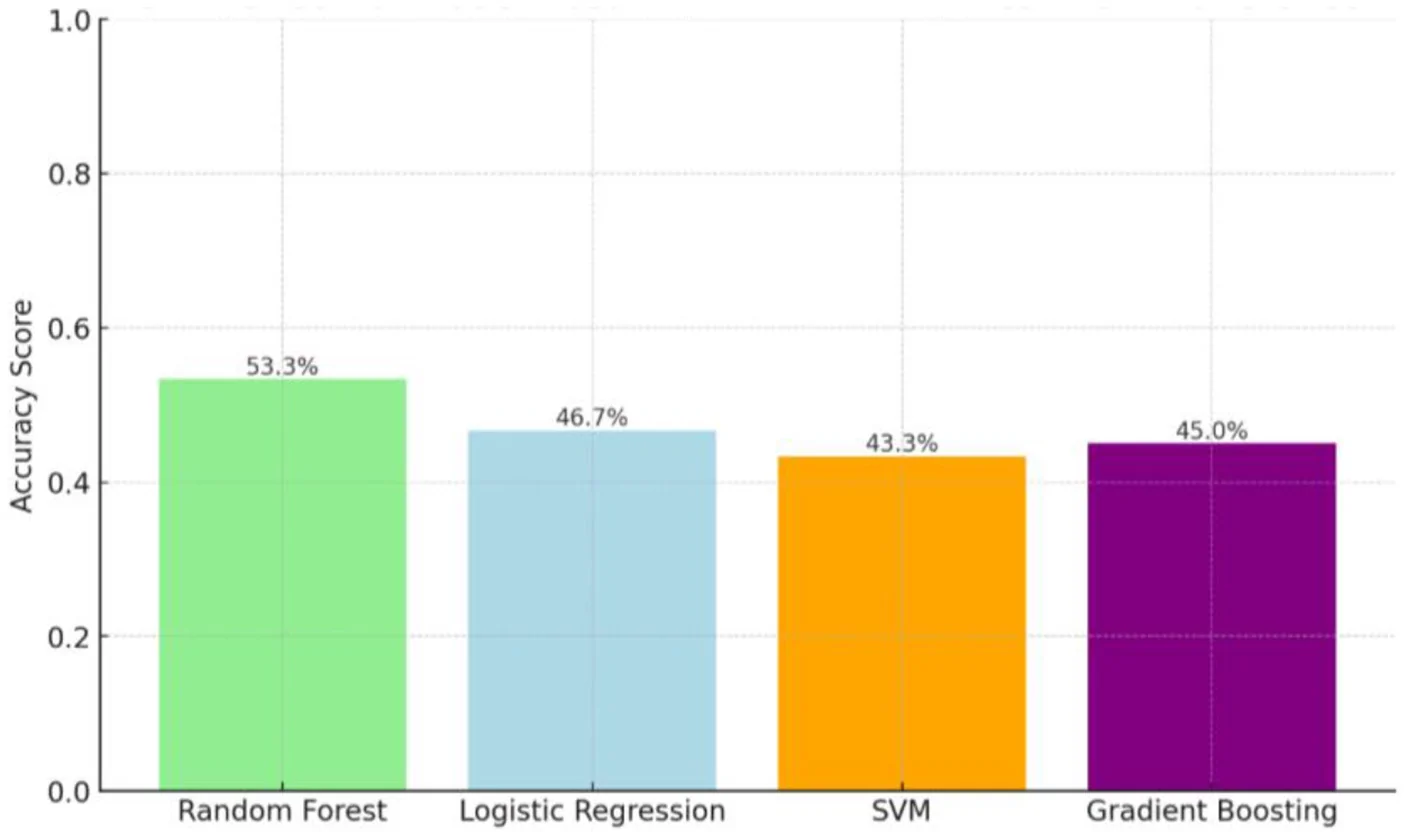
Raw accuracy (%) and balanced accuracy (%) of Random Forest, Logistic Regression, Gradient Boosting, and Support Vector Machine (SVM) compared with the naïve baseline in classifying DR-TB treatment adherence. a = Models trained on 80% of the dataset and tested on 20% (hold-out validation). b = Accuracy values are descriptive; no formal statistical comparison between models was performed due to limited sample size.

Among the four models tested, the Random Forest algorithm achieved the highest overall accuracy (53.3%), outperforming logistic regression (46.7%), gradient boosting (45.0%), and SVM (43.3%) (Table 3, Figure 4). Accuracy represents the proportion of all predictions, both adherent and non-adherent, that were correct. Random Forest also demonstrated the best balance between precision and recall, as reflected in its highest F1 score (0.59). In this context, precision measures the proportion of patients the model identified as adherent who were truly adherent, while recall measures the proportion of all truly adherent patients correctly detected by the model. The F1 score, being the harmonic mean of precision and recall, provides a single summary of this balance, which is important in DR-TB care because both missing a high-risk patient (low recall) and flagging too many low-risk patients unnecessarily (low precision) can have serious consequences for resource allocation and patient outcomes. These findings suggest that while the overall predictive performance was modest, the Random Forest model offered the most clinically balanced trade-off between identifying at-risk patients and avoiding excessive false alerts, making it the most promising approach in this comparison for guiding targeted adherence interventions.

**Table 3:** Comparison of model accuracy, precision, recall, and F1-score across four machine learning algorithms predicting DR-TB treatment adherence (*n* = 108).

**Model**	**Raw accuracy (%)**	**Balanced accuracy (%)**	**Precision**	**Recall**	**F1-Score**
Random forest	81.5	53.3	0.61	0.58	0.59
Logistic regression	79.6	50.0	0.52	0.47	0.49
Gradient boosting	78.7	49.5	0.51	0.45	0.47
Support vector machine (SVM)	77.8	48.7	0.49	0.43	0.45
Naïve baseline	79.6	50.0	0.796	1.000	0.887

Raw accuracy (%) = proportion of all predictions (adherent and non-adherent) that were correct.

Precision = proportion of patients predicted to be adherent who were truly adherent.

Recall = proportion of truly adherent patients correctly identified by the model.

F1-score = harmonic mean of precision and recall, balancing false positives and false negatives.

## 4 Discussion

This study investigated the predictors of treatment adherence among patients with DR-TB in a rural South African setting, focusing on socioeconomic, demographic, and clinical factors. Using a Random Forest model, we found that age, education, income, patient category, and social history were the most influential factors in adherence classification. These findings contribute to the growing evidence that treatment adherence is influenced not only by individual health but also by broader social and economic factors (5, 8, 16, 28, 29).

### 4.1. Interpretation of key findings

Our results indicate that younger patients generally achieve higher adherence rates than older adults. Adherence to medication and treatment regimens varies by age, but the relationship is complex and depends on the disease, treatment type, and patient population. Some research findings indicate that older adults often have equal or better adherence rates than younger adults, contrary to common assumptions. A Singapore study's findings documented a contrary result to our findings with medication non-adherence in young adults (38.4%), higher than that in older adults (22.3%). Reasons given for non-adherence in young adults were mainly patient- and therapy-related factors, which were generally identical to those identified in older adults (30). Other studies conducted in Singapore and Japan reported a higher level of adherence among a convenient sample of community-dwelling adults (31, 32). Adherence is generally higher in older adults, but can decline with cognitive impairment, depression, or very advanced age (>80) (33). While some studies suggest older adults may adhere better due to greater health awareness, our data indicate that, in this rural setting, older age is more often associated with challenges such as comorbidities, physical limitations, and treatment fatigue. These barriers may be compounded by the complexity of managing DR-TB alongside HIV and other non-communicable diseases (1, 34, 35). This highlights the need for age-tailored interventions, for example, simplifying regimens, involving caregivers, and improving community health worker follow-up to sustain adherence in older patients.

Education level and income emerged as strong positive predictors of adherence, aligning with previous findings that socioeconomic stability facilitates better health behaviors (29, 36). According to the study carried out in Malaysia, the odds of non-adherence were higher among participants with no formal education in comparison with those who have (37) Studies carried out in other regions such as in Southwest Ethiopia (38) southeast Nigeria (39), Republic of Korea (36), and Indonesia (40) support the same findings. Nonetheless, with other studies, no association was found between education and adherence to anti-TB drugs (41, 42). In our context, higher education likely enhances understanding of treatment importance, reducing misconceptions and increasing motivation, while higher income may improve access to transport, nutritious food, and ancillary care. However, these associations should be understood as markers of underlying structural inequalities. Addressing adherence, therefore, requires interventions that go beyond individual counseling to include socioeconomic support, such as travel stipends, nutritional assistance, and workplace flexibility for employed patients (43, 44).

Social and behavioral factors, particularly peer influence, alcohol use, and smoking, were associated with reduced adherence. This is strongly supported by Garbrah et al. (45) and Nezenega et al. (46), who reported a reduced likelihood of treatment adherence with patients who used substances such as alcohol, marijuana, and cigarettes. Peer influence had the strongest negative impact, consistent with TB adherence studies from Ethiopia and Ghana (44, 45). It reflects how social networks can either support or hinder treatment motivation (47, 48). In rural communities with high TB stigma, negative peer messaging can discourage clinic attendance. Behavioral health interventions that integrate mental health counseling, peer-support groups, and substance-use programmes may be critical to mitigating these risks (28, 49).

### 4.2. Predictive modeling and clinical implications

Random Forest models proved superior in predicting treatment adherence in our dataset, outperforming logistic regression, gradient boosting, and support vector machines (SVMs) with an accuracy of 53.3%. This result highlights the model's key advantage: its capacity to effectively capture non-linear relationships and interactions between complex socioeconomic and clinical variables.

While studies in other health domains have reported significantly higher accuracies (e.g., up to 0.82–0.99), these results should be interpreted with caution. The high accuracy in those contexts often pertains to different prediction tasks, such as disease diagnosis from images or biomarkers, and may not be directly applicable to the behavioral complexities of TB treatment adherence. The modest accuracy of our model, while superior to other approaches in our specific dataset, underscores the inherent difficulty in predicting a variable as complex and multidimensional as patient adherence (50). Age, education, and income accounted for the largest proportion of predictive importance, followed by patient category and social history. While the model's accuracy was modest, it demonstrates potential as a clinical decision-support tool for early identification of high-risk patients. Random Forest outperformed traditional statistical models and other machine learning methods in our dataset. Although high accuracies for Random Forest have been reported in other health domains (51), these findings may not directly generalize to TB adherence prediction. Integrating Random Forest-based predictions into electronic medical records could allow healthcare providers to target adherence counseling and social support resources more efficiently by focusing interventions on high-risk patients and potentially improving overall adherence rate (50–52). While Random Forest models show strong performance in retrospective and specific clinical datasets, their effectiveness may vary across populations and settings, necessitating local validation. Hence, these findings must be interpreted with caution. The absence of an external validation dataset means that the model's performance in other regions remains untested. Variables such as “peer influence” and locally derived income categories were defined contextually and may not exist in a comparable form in public datasets. Future work should prioritize assembling harmonized, multi-site datasets to enable robust external validation and to test whether Random Forest, or alternative algorithms, maintain performance across diverse epidemiological and social settings.

### 4.3. Comparison with existing literature

Adherence rates in our cohort (79.6%) are higher than those reported in some DR-TB studies from Ethiopia (37, 38) and South Korea (1) but slightly lower than the Kosovo findings (53). These differences likely reflect variations in treatment protocols, adherence measurement, and health system support. Unlike many studies that assess adherence through single measures such as pharmacy refill or self-report, our operational definition integrated treatment duration against an ≥80% threshold, consistent with WHO guidance (8, 9, 54). This standardization allows for more meaningful comparison but also underscores the need for uniform reporting in global DR-TB research. Our analysis adds depth to existing literature by linking socio-behavioral variables, particularly peer influence, to modeled adherence risk in a rural African setting. Few DR-TB predictive modeling studies have incorporated such qualitative-derived variables, and their inclusion here demonstrates that even “non-traditional” predictors can meaningfully improve model interpretability.

### 4.4. Implications for policy and practice

The findings underscore that improving DR-TB adherence in rural South Africa requires a multifaceted strategy that combines targeted support for older adults through age-specific interventions, socioeconomic measures to address transport, nutrition, and employment-related barriers, and behavioral health integration to mitigate the negative effects of peer pressure, alcohol use, and smoking. Incorporating predictive risk stratification can further enable the identification and prioritization of high-risk patients for intensified support. Together, these priorities align with the WHO End TB Strategy's focus on integrated, patient-centered care (8, 44) while extending its application by operationalising locally specific social factors within predictive modeling. This combined approach linking structural support with precision targeting offers a practical pathway for optimizing resource allocation and improving outcomes in high-burden, resource-limited settings.

## 5 Limitations of study and future directions

This study highlights key factors influencing DR-TB management in the rural Eastern Cape, where poverty and socioeconomic challenges complicate adherence. Limitations include potential recall bias from self-reported data, inconsistent documentation of mental health conditions, and reliance on qualitative notes to assess “peer influence” without standardized tools. Model comparisons were descriptive only due to the small sample size, and no external validation was performed because comparable datasets with equivalent variables were unavailable. Variables like “peer influence” and locally derived income categories were context-specific and may not exist in other datasets, limiting direct transferability. While Random Forest performed best in this setting, its utility elsewhere would require retraining with local data. Income was inferred from employment status rather than recorded as an exact numeric value, introducing the possibility of misclassification. For example, informal sector workers may have variable earnings that do not align with the assumed “low income” category, and some government employees may earn outside the typical middle-income range. These limitations may have attenuated the observed association between income and adherence. Future studies should collect direct, self-reported income or validated SES indices to improve measurement accuracy. Furthermore, structured psychosocial assessments, integrated mental health screening, larger multi-site datasets, and robust external validation should be included in predictive modeling to improve its generalisability and predictive accuracy.

## 6 Recommendation and conclusion

Tailored Support: Interventions should focus on older patients and those with low formal education. This includes providing simplified, culturally appropriate health education materials using visual aids and engaging family or caregivers. Integrated Care: it is crucial to integrate support for patients managing multiple health conditions, such as DR-TB, HIV (ART), and non-communicable diseases (NCDs). Socioeconomic Support: the study aligns with WHO recommendations by stressing the importance of comprehensive patient support, including adherence counseling, transportation stipends, and food packages. These measures are essential for addressing socioeconomic barriers. Predictive Tools: using predictive models like the Random Forest model can help identify high-risk individuals early, allowing for personalized, proactive care. Collaborative Approach: addressing the stigma associated with TB and sustaining adherence requires collaborative efforts across health sectors and community structures.

## Data Availability

The raw data supporting the conclusions of this article will be made available by the authors, without undue reservation.
